# New clinical insights into the treatment of benign uretero‐ileal anastomotic stricture following radical cystectomy and urinary diversion

**DOI:** 10.1002/cam4.70229

**Published:** 2024-09-13

**Authors:** Yixuan Mou, Cenchao Yao, Zhenghong Liu, Pu Zhang, Xiaolong Qi, Dahong Zhang, Yiyang Chen, Weiwen Yu, Shuai Wang

**Affiliations:** ^1^ Urology and Nephrology Center, Department of Urology Zhejiang Provincial People's Hospital, Affiliated People's Hospital, Hangzhou Medical College Hangzhou China

**Keywords:** benign uretero‐ileal anastomotic stricture, bladder cancer, endoscopic treatment, radical cystectomy, robotic and laparoscopic ureteral reimplantation

## Abstract

**Background:**

Benign uretero‐ileal anastomotic stricture (UIAS) is a potentially serious complication that can arise after radical cystectomy (RC) and subsequent urinary diversion. To preserve residual renal function and improve prognosis, it is crucial to derive insights from experience and tailor individualized treatment strategies for different patients.

**Patients and Methods:**

From October 2014 to June 2021, a total of 47 patients with benign UIAS underwent endoscopic management (*n* = 19) or reimplantation surgery (*n* = 28). The basic data, perioperative conditions, and postoperative outcomes of the two groups were compared and analyzed to evaluate efficacy.

**Results:**

Comparing preoperative and postoperative clinical efficacy within the same group, the endoscopic group showed no significant differences in creatinine and blood urea nitrogen (BUN) levels before surgery or after extubation (*p* > 0.05). However, significant differences were observed in glomerular filtration rate (GFR) levels on the affected side before surgery and after extubation (*p* < 0.05). In contrast, the laparoscopic reimplantation group did not exhibit significant differences in creatinine, BUN, or GFR levels of affected side before surgery and after extubation (*p* > 0.05). Postoperative clinical efficacy showed no significant difference in creatinine and BUN levels between the two groups (*p* > 0.05). However, GFR values of affected side in the endoscopic treatment group decreased more than those in the laparoscopic reimplantation group (*p* < 0.05). Additionally, the laparoscopic reimplantation group was able to remove the single‐J tube earlier than the endoscopic treatment group (*p* < 0.05), had a lower recurrence rate of hydronephrosis after extubation (*p* < 0.05), and experienced a later onset of hydronephrosis compared to the endoscopic treatment group (*p* < 0.05).

**Conclusions:**

Based on our experience in treating UIAS following RC combined with urinary diversion, laparoscopic reimplantation effectively addresses the issue of UIAS, allowing for the removal of the ureteral stent relatively soon after surgery. This approach maintains long‐term ureteral patency, preserves residual renal function, reduces the risk of ureteral restenosis and hydronephrosis, and has demonstrated superior therapeutic outcomes in this study.

## 1 INTRODUCTION

Radical cystectomy (RC) combined with urinary diversion (UD) is recognized as the gold standard and an effective treatment for high‐risk non‐muscle‐invasive bladder cancer (NMIBC) and muscle‐invasive bladder cancer (MIBC) confined to the organs.^1^ Among UD methods, ileal conduit is the most commonly performed due to its simplicity, while orthotopic neobladders is preferred for optimal preservation of intact natural urinary tract. Continent cutaneous diversions also present a viable alternative, with both continent and incontinent diversions being critical for the prognosis of bladder cancer.^2^, ^3^ However, to further enhance prognosis, certain postoperative complications, such as gastrointestinal issues (e.g., diarrhea and ischemic intestine perforation), genitourinary complications (e.g., ureterolithiasis and anastomotic stricture), and herniation (e.g., hernia and parastomal hernia), still need to be addressed.^4^ In these complications, postoperative benign uretero‐ileal anastomotic stricture (UIAS) is a significant complication, with an incidence ranging from 3% to 30%.^5^, ^6^, ^7^, ^8^ If not promptly treated, UIAS can lead to complications such as pyelonephritis, hydronephrosis, stone formation, and renal impairment.^9^ Richards et al.^10^ identified the common causes of UIAS as anastomotic fibrosis, inflammation, and tumor recurrence, with fibrosis being the most challenging factor to manage. Thus, it is crucial to compare and analyze different treatment approaches for UIAS and to develop individualized treatment strategies tailored to each patient's needs.

Treatment options for benign UIAS can be broadly categorized into surgical and endoscopic approaches. Surgical methods include open and laparoscopic ureteral reimplantation, using either conventional or robot‐assisted techniques.^11^ Endoscopic interventions include balloon dilation and incision, using techniques like ureteroscopic balloon dilation and endoscopic incision with holmium laser, monopolar instruments, or a cold knife.^12^ Advancements in endoscopic techniques have made it becoming a preferred initial treatment option. The decision between surgical and endoscopic approaches is influenced by factors such as the severity of the stricture, patient anatomy, and the surgeon's expertise.^13^ Each method has its own benefits, and the choice is typically tailored to the unique characteristics of each individual case.

Since October 2014, our department has implemented various treatment modalities for benign UIAS following RC combined with UD, including endoscopic surgery (balloon dilation or holmium laser incision) and ureteral reimplantation (robotic or conventional laparoscopic). Laparoscopic ureteral reimplantation is recognized for its high success rate but is limited by its complexity and the potential for surgical trauma. Conversely, endoscopic surgery is considered less invasive and safer, although its effectiveness requires further investigation.^14^ With the availability of multiple treatment options, selecting the optimal approach for managing benign UIAS has become a critical issue. To provide more evidence for making informed decisions, we conducted a retrospective analysis of patient data, comparing the efficacy of these two approaches after the initial diagnosis of benign UIAS.

## 2 PATIENTS AND METHODS

### 2.1 Patient Information

Following approval from the Institutional Review Board, we retrospectively reviewed the medical records of patients who developed benign UIAS after RC combined with UD (either ileal conduit or orthotopic neobladder) at Zhejiang Provincial People's Hospital. All procedures adhered to relevant guidelines and regulations. From October 2014 to June 2021, a total of 47 patients with benign UIAS were identified and divided into two groups based on their management approach: endoscopic management (Group A) and laparoscopic reimplantation (Group B). All patients' preoperative biochemical tests, renal function assessments, imaging studies, and follow‐up data were collected.

### 2.2 Study methods

Patient demographics are detailed in Table 1. Preoperative radiographic imaging (CT urography, ultrasound, and renal emission computed tomography (ECT)), was conducted to confirm the diagnosis and assess the stricture's length and location. Anastomotic stricture was defined as the radiographically diagnosed hydronephrosis.^14^ If renal function did not improve with conservative treatment, prompt surgical intervention was recommended to relieve the obstruction. Previous research has indicated that endoscopic balloon dilation is highly effective for benign ureteral strictures with a length of ≤2 cm and onset ≤3 months.^15^ In this study, the choice of surgical approach was based on stricture length and patient consent. Hydronephrosis severity was classified based on preoperative CT urography into three grades:

1. Mild hydronephrosis: normal renal parenchyma thickness with renal collecting system separation of 2–3 cm;
2. Moderate hydronephrosis: separation of the renal collecting system of approximately 3–4 cm, with dilated pelvis and calyces and thinned renal parenchyma;
3. Severe hydronephrosis: thinned renal cortex and separation of the renal collecting system greater than 4 cm.

**TABLE 1:** Case characteristics.

Overall	Group A *N* = 19 (%)	Group B *N* = 28 (%)	*p* Value
Sex			
M	16 (84.2)	25 (89.3)	0.947^a^
F	3 (15.8)	3 (10.7)	
Mean age (X ± S years)	70 ± 11	72 ± 10	0.486
BMI (X ± S kg/m^2^)	22.7 ± 2.6	22.6 ± 2.5	0.985
Duration time (months)	11.0 ± 3.0	12.0 ± 3.5	0.693
Side affected			
Left	8 (42.1)	14 (50.0)	
Right	8 (42.1)	10 (35.7)	
Bilateral	3 (15.8)	4 (14.3)	
Stricture length (cm)			
≤1	15	16	
>1	4	12	
Urinary diversion			
Bricker	12	17	
In situ	7	11	
Creatinine (X ± S μmol/L)	107 ± 27	114 ± 37	0.475
Urea (X ± S mmol/L)	8.6 ± 2.8	9.1 ± 4.1	0.632
Affected side GFR (X ± S mL/min)	24.1 ± 2.9	24.7 ± 3.3	0.544
Hb (X ± S g/mL)	117 ± 20	118 ± 18	0.855
Degree of hydronephrosis			
Mild	15 (78.9)	19 (67.9)	0.888^b^
Moderate	3 (15.8)	5 (17.9)	
Severe	1 (5.3)	3 (10.8)	

^a^ *p* value—continuity correction method.

^b^ *p* value—Fisher's exact probability method.

Overall, 47 patients underwent treatment for benign UIAS, with 19 receiving endoscopic management and 28 undergoing reimplantation surgery. The endoscopic management group included techniques such as endoscopic balloon dilation^16^, ^17^ and endoscopic holmium laser incision.^18^ For balloon catheter dilation in this study, a combined anterograde and retrograde dilation approach was employed. The procedure involved a puncture being made under ultrasound guidance, before a guide wire was introduced. The skin and fascia were cut using a small pointed knife guided by the guide wire. Based on the guide wire, a fascia expander was used to dilate the channel toward the renal pelvis to a diameter of 22F. A 22Fr skinning sheath was then placed, the expander withdrawn, and a ureteroscope introduced. The distal ureteral stricture was visualized, and another guide wire was inserted retrogradely through the ileal stoma to access the ureteroscope. This guide wire's position was used to evaluate the stricture at the ureteral anastomosis. The ureteroscope was then advanced along the guide wire for a detailed examination of the stenosis at the anastomosis site. To address the narrowing, an Olbert balloon dilator (7‐Fr) was inserted through the first guide wire, inflated with water to 25 atmospheres, and maintained for approximately 5 min to dilate the constricted segment of the ureteral anastomosis. After dilation, the ureteroscope was reintroduced to inspect the anastomosis, and an Fr6 single‐J stent was placed. The guide wire and ureteroscope were then withdrawn, and the skin was sutured. Postoperatively, patients were closely monitored for bleeding, infection, and other symptoms. The nephrostomy tube was removed following a computed tomography (CT) review.

**FIGURE 1:**
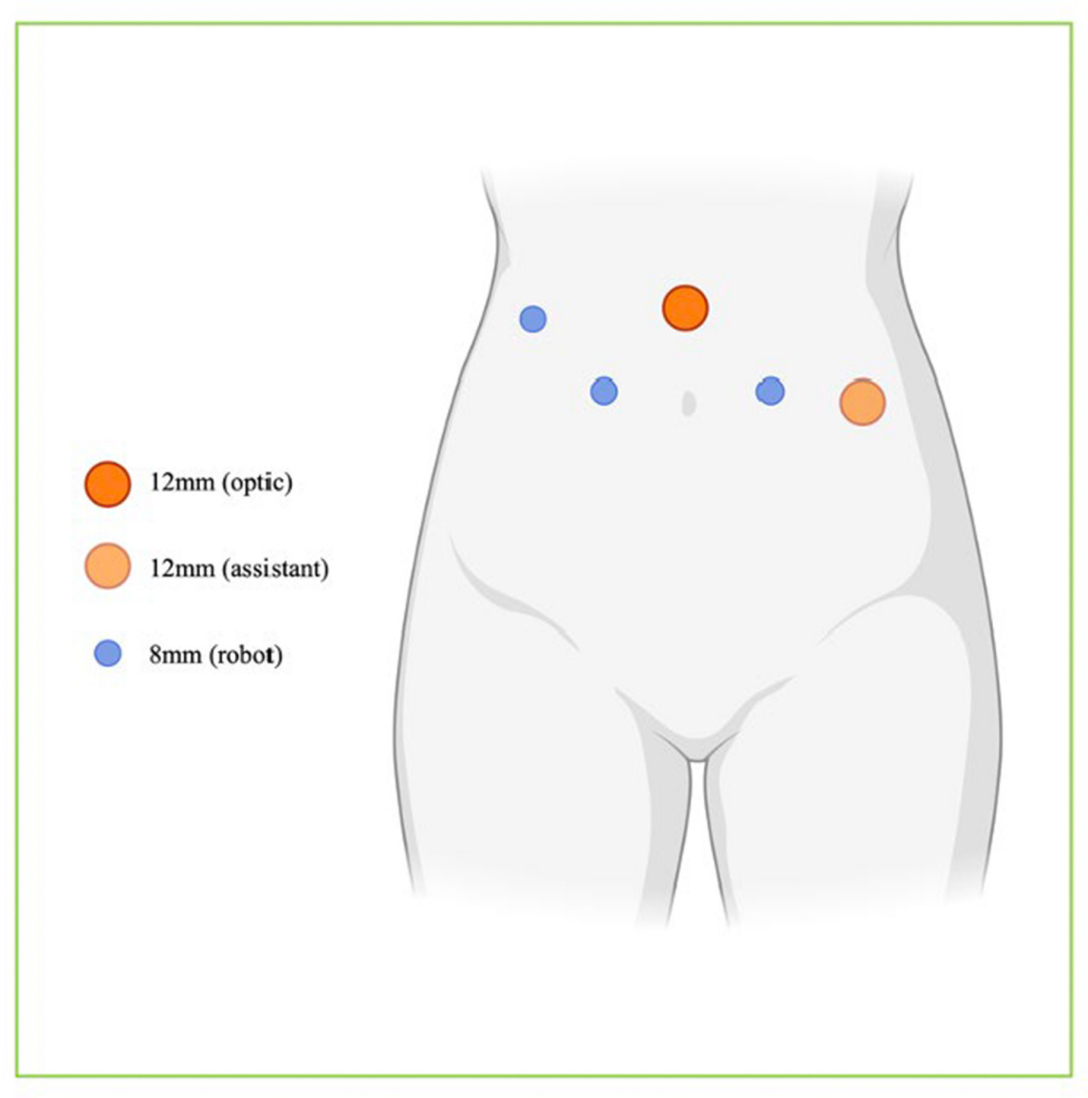
Port placement of robot‐assisted laparoscopic ureteral replantation.

**FIGURE 2:**
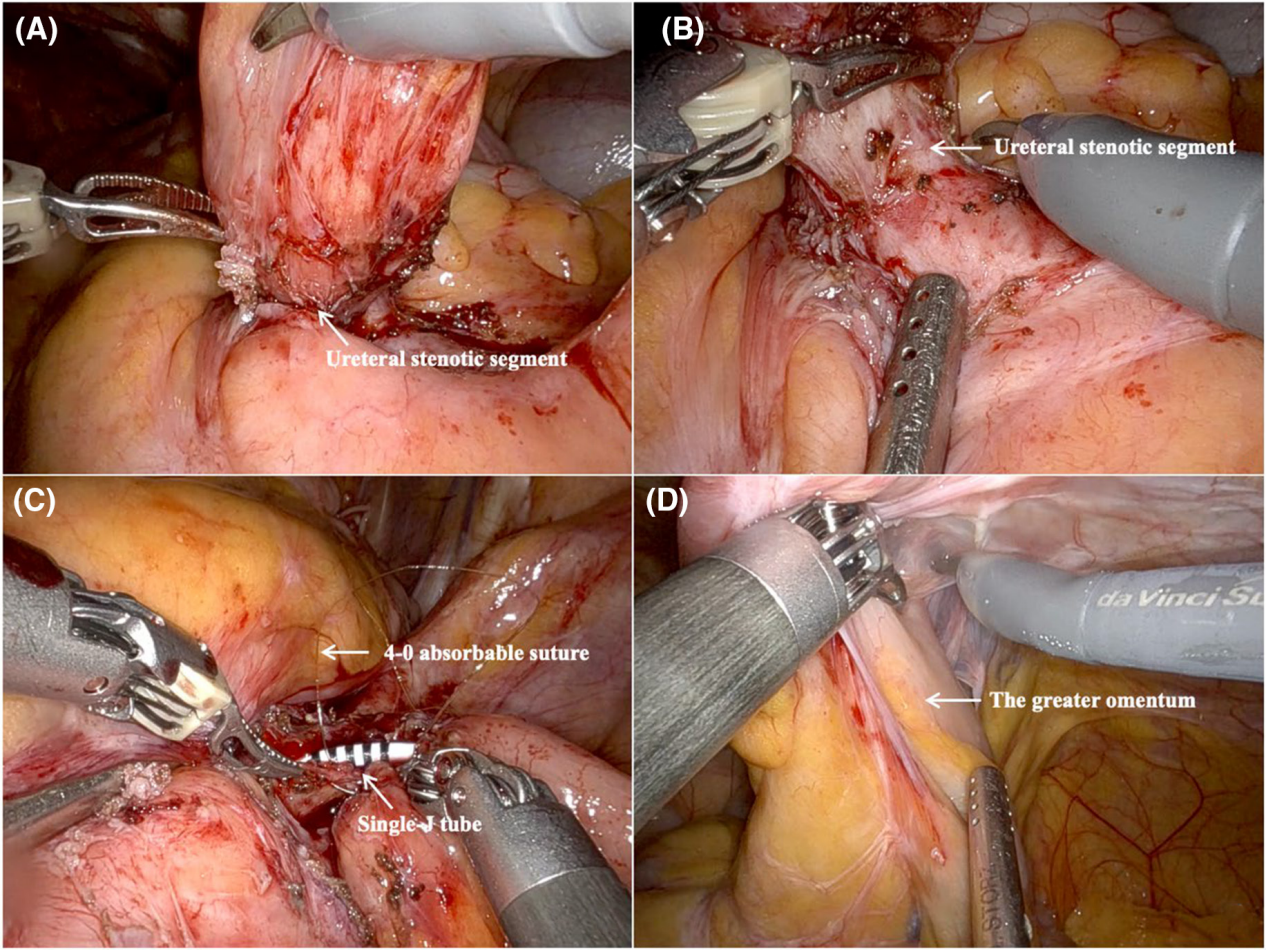
Robot‐assisted laparoscopic ureteral replantation. Separated the ureter carefully to the outflow intestinal segment, the lower ureteral wall was seen to be stiff and luminal narrowed (A, B); a single‐J tube was placed between the ureteral stenotic side and the new segment of the outflow tract (C); dissociated a portion of the greater omentum (D).

The laparoscopic reimplantation group was divided into two subgroups: robot‐assisted laparoscopic ureteral reimplantation (*n* = 18)^19^ and conventional laparoscopic ureteral reimplantation (*n* = 10).^20^ Trocars were placed as follows: a 12‐mm trocar at the left anterior axillary line flat umbilicus and 8 mm trocars at the right anterior axillary line flat umbilicus, the left midclavicular line flat umbilicus, and the right midclavicular line flat umbilicus, as illustrated in Figure 1. The dilated ureter on the stenotic side was anastomosed to a new segment of the ileal conduit using interrupted 4–0 absorbable sutures, ensuring a tension‐free and non‐torsional connection. A single‐J stent of Fr6 was placed. Additionally, a segment of the greater omentum was dissected to cover and secure the lower part of the ureter on the stenotic side, providing adequate blood supply, as depicted in Figure 2.

Subsequently, perioperative data were carefully collected, including preoperative values for creatinine, urea, and GFR on the affected side, as well as intraoperative bleeding, postoperative hospitalization duration, postoperative feeding time, and whether intraoperative or postoperative blood transfusions were required. ECT was performed on all patients before and after surgery to assess unilateral GFR, with comparisons limited to the affected side in this study. A single‐J stent was conventionally maintained for 3 months post‐surgery, in line with periodic stent replacement recommendations.^21^ After this period, a urinary tract CT was performed to assess the condition. If hydronephrosis was still present, the stent was replaced endoscopically. If hydronephrosis persisted after the stent's routine removal, the single‐J stent was reinserted.

### 2.3 Data analysis

Statistical analyses were performed using SPSS 26.0 software. Continuous variables were assessed using the Student *t*‐test, while categorical data were analyzed with the Fisher exact test. A *p*‐value of <0.05 was considered statistically significant.

## 3 RESULTS

### 3.1 Perioperative data

There were no significant differences in gender, age, body mass index (BMI), preoperative creatinine, blood urea nitrogen (BUN), glomerular filtration rate (GFR), or hemoglobin (Hb) levels between the two groups (*p* > 0.05). Additionally, preoperative imaging showed no significant differences in the degree of hydronephrosis between the groups (*p* > 0.05). Significant differences were observed between the two groups in surgical time, intraoperative bleeding volume, postoperative hospital stay, and postoperative feeding time (all *p* < 0.05). However, for blood transfusions, Group A had no cases with transfusion, while Group B had two cases with transfusion. No statistically significant difference was found between the two groups (*p* > 0.05) regarding transfusion rates. Detailed data are provided in Table 2.

**TABLE 2:** Clinical efficacy comparison.

	Group A *N* = 19	Group B *N* = 28	*p* Value
Operative time (X ± S min)	96 ± 35	184 ± 39	0.000
Bleeding volume (X ± S mL)	17 ± 11	84 ± 41	0.000
Postoperative hospitalization (X ± S days)	5.8 ± 1.8	8.4 ± 3.3	0.004
Postoperative feeding time (X ± S days)	1.7 ± 0.9	2.9 ± 1.3	0.001
Perioperative blood transfusion			
No blood transfusion	19 (100)	26 (92.9)	0.650^a^
Blood transfusion	0	2 (7.1)	
Preoperative			
Creatinine (X ± S μmol/L)	107 ± 27	114 ± 37	0.105
Urea (X ± S mmol/L)	8.6 ± 2.8	9.1 ± 4.1	0.070
Affected side GFR (X ± S mL/min)	24.1 ± 2.9	24.7 ± 3.3	0.000
Postoperative			
Creatinine (X ± S μmol/L)	102 ± 20	106 ± 28	0.118
Urea (X ± S mmol/L)	7.9 ± 1.7	8.1 ± 3.2	0.108
Affected side GFR (X ± S mL/min)	17.9 ± 3.2	24.2 ± 3.5	0.107
Extraction time of ureteral stent (mouths)	6.5 ± 2.2	4.2 ± 3.8	0.043
Ureteral stent in place	4	2	
Hydronephrosis after extubation			
No	7	21	0.009^b^
Yes	12	7	
Time of hydronephrosis after extubation (months)	5.4 ± 2.7	11.8 ± 1.9	0.000

^a^ *p* value—continuity correction method.

^b^ *p* value—Pearson card method.

### 3.2 Clinical efficacy analysis

#### 3.2.1 Intra‐group comparison

Creatinine, urea, and GFR are crucial indicators for evaluating renal function and evaluating the efficacy of the two treatment modalities. In Group A, no statistically significant differences were found in creatinine and urea values before and after endoscopic treatment (*p* > 0.05). However, there was a significant change in GFR on the affected side before and after surgery (*p* < 0.05). This indicates that while creatinine and urea values did not significantly decrease following endoscopic treatment, the GFR on the affected side decreased, suggesting that endoscopic treatment did not markedly improve renal function post‐surgery, and renal function on the affected side continued to decline.

In Group B, the postoperative creatinine, urea, and GFR levels on the affected side did not exhibit significant changes after laparoscopic reimplantation (*p* > 0.05). This suggests that laparoscopic reimplantation may help salvage residual renal function and alleviate hydronephrosis to some extent. The absence of a significant decrease in overall creatinine and urea values could be due to the fact that renal function on the affected side was not severely compromised, with the contralateral kidney potentially compensating for any functional decline.

#### 3.2.2 Inter‐group comparison

Clinical efficacy was evaluated by comparing preoperative and postoperative values of creatinine, urea, and GFR, as well as the extubation time and recurrence of hydronephrosis between the two groups. Postoperative imaging is often used to assess the recurrence of stenosis or hydronephrosis after surgery. The comparison of *D*‐values for creatinine and urea between the two groups did not reveal a statistically significant difference (*p* > 0.05). However, the *D*‐value in GFR on the affected side did show a statistically significant difference (*p* < 0.05). Although creatinine and urea levels did not differ significantly between the groups, the endoscopic treatment group experienced a more significant decrease in GFR values compared to the laparoscopic reimplantation group, indicating a continued decline in renal function. Additionally, there was a statistically significant difference in the extraction time of the single‐J tube between the two groups (*p* < 0.05). Follow‐up via telephone and electronic medical records revealed that four patients in Group A and two patients in Group B still had the single‐J tube in place. Furthermore, the observation of hydronephrosis after extubation and the timing of its occurrence showed a significant difference between the two groups (*p* < 0.05). Overall, while creatinine and urea levels remained similar between the groups, the endoscopic treatment group exhibited a greater decline in GFR, longer retention of the single‐J tube, and a higher incidence of recurrent hydronephrosis after extubation compared to the laparoscopic reimplantation group.

The results essentially show that endoscopic treatment offers only temporary relief from hydronephrosis and does not provide a long‐term solution for ureteral stenosis, leading to continued decline in renal function on the affected side and a high incidence of restenosis. Conversely, laparoscopic reimplantation proves to be a more effective approach for maintaining ureteral patency, preserving residual renal function, alleviating hydronephrosis, and reducing the incidence of restenosis.

## 4 DISCUSSION

Complications following RC combined with UD are reported to have an incidence ranging from 25% to 35%.^22^ Benign UIAS is a relatively common complication, with an incidence reported to range from 2.7% to 10% and a median diagnosis time of 7–18 months post‐surgery.^23^ Patients with benign UIAS may either be asymptomatic or present with mild symptoms such as obstruction, infection, or stone formation.^7^, ^24^ Diagnosis is often triggered by changes in renal function. Without timely intervention, anastomotic closure can result in renal hydronephrosis and irreversible renal function decline.^25^ Wang et al.^25^ highlighted that specific surgical techniques, such as passing the left ureter through the sigmoid colon mesentery to achieve sufficient length for anastomosis with the neobladder, may increase the incidence of left UIAS. Therefore, precise surgical techniques—such as careful ureteral disconnection, appropriate tissue separation, minimized interruption of ureteral blood supply, and tension‐free anastomosis—are crucial for reducing the risk of benign UIAS. The surgeon's expertise and the use of robotic assistance significantly contribute to successful outcomes. However, a direct comparison between robotic and laparoscopic reimplantation was not performed due to the limited sample size.

Minimally invasive surgery and laparoscopic ureteral reimplantation are currently the leading treatment options for benign UIAS globally.^26^ Minimally invasive approaches include cystoscopic retrograde intubation, antegrade percutaneous nephroureteroscopy combined with retrograde cystoscopic dilation, and endoscopic holmium laser incision. These techniques generally result in fewer complications and a shorter recovery period compared to open surgery.^17^ However, some experts advocate that the gold standard for treating benign UIAS is to excise the narrowed segment and reimplant the ureter.^27^ Based on our experience, secondary surgical treatment is often complicated by disrupted anatomical structures due to the initial surgery. A considerable portion of the procedure time is spent resolving tissue issues and freeing abdominal adhesions. Additionally, locating the anastomosis and the narrowed segment between the ileal bladder and ureter can be challenging, increasing the difficulty of the procedure and the incidence of complications. Therefore, a thorough study and comparison of these two treatment methods are essential to determine the most effective approach.

In this study, there was no significant statistical difference between the two groups regarding gender, average age (70 years ± 11 years vs. 72 years ± 10 years), BMI (22.7 ± 2.6 vs. 22.6 ± 2.5), preoperative creatinine levels (107 ± 27 vs. 114 ± 37) μmol/L, preoperative urea levels (8.6 ± 2.8 vs. 9.1 ± 4.1) mmol/L, preoperative GFR on the affected side (24.1 ± 2.9 vs. 24.7 ± 3.3) mL/min, and the degree of hydronephrosis (*p* > 0.05). This indicates that the baseline data for the two groups were comparable and suitable for further analysis.

Regarding intraoperative data, there were statistically significant differences (*p* < 0.05) between Group A and Group B in terms of surgical time (96 ± 35 vs. 184 ± 39) mins, intraoperative bleeding (17 ± 11 vs. 84 ± 41) days, postoperative hospital stay (5.8 ± 1.8 vs. 8.4 ± 3.3) days, and postoperative feeding time (1.7 ± 0.9 vs. 2.9 ± 1.3) days. Compared to laparoscopic reimplantation, endoscopic treatment demonstrated acceptable immediate success with fewer complications and lower morbidity,^28^ and offered advantages such as shorter surgical time, reduced bleeding, better safety, and quicker postoperative recovery.

Based on the postoperative data, comparing biochemical test values during postoperative hospitalization may not accurately reflect the treatment's efficacy. Therefore, we assessed surgical outcomes using imaging examinations, biochemical tests, and GFR measurements on the affected side after extubation. The preoperative and postoperative creatinine, urea, and GFR values of each group were compared. The results showed no significant statistical difference (*p* > 0.05) in creatinine levels (107 ± 27 vs. 102 ± 20) μmol/L and urea levels (8.6 ± 2.8 vs. 7.9 ± 1.7) mmol/L in the endoscopic treatment group, but a statistically significant difference (*p* < 0.05) in GFR values on the affected side (24.1 ± 2.9 vs. 17.9 ± 3.2) mL/min. This indicates that patients treated with endoscopy did not experience significant improvement in renal function after extubation, with some even developing ureteral restenosis and hydronephrosis, leading to a continued decline in renal function. A large‐scale study involving 697 patients reported a success rate of 46% for endoscopic surgery, with higher success rates for strictures ≤1 cm and right‐sided strictures. The success rate in our study was similar (37%); however, the previous study did not compare postoperative renal function indicators.^29^

The creatinine value (114 ± 37 vs. 106 ± 28) μmol/L, urea value (9.1 ± 4.1 vs. 8.1 ± 3.2) mmol/L, and GFR value on the affected side (24.7 ± 3.3 vs. 24.7 ± 3.3) mL/min in the laparoscopic reimplantation group showed no significant statistical difference (*p* > 0.05). This suggests that there were no significant changes in creatinine, urea, or GFR values on the affected side after extubation in the laparoscopic reimplantation group. This may be due to the fact that the renal function on the affected side of most patients did not experience significant deterioration, and the contralateral kidney remained intact, providing compensatory function. Essentially, we found that laparoscopic reimplantation can improve or slow the deterioration of renal function to some extent and provide long‐term relief from hydronephrosis. Moreover, for patients with bilateral stenosis, laparoscopic reimplantation can address both sides concurrently, whereas endoscopic treatment typically involves surgery on one side with temporary nephrostomy on the other, necessitating a second surgery.

The placement of a single‐J tube is essential for ensuring ureteral patency after surgery, and evaluating the patency and function of the ureter post‐extubation is key to assessing the effectiveness of different treatment methods. In this study, there were statistically significant differences (*p* < 0.05) between the two groups regarding the time to extubation (6.5 ± 2.2 vs. 4.2 ± 3.8) months, incidence of hydronephrosis after extubation, and the time to onset of hydronephrosis post‐extubation (5.4 ± 2.7 vs. 11.8 ± 1.9) months. Compared to endoscopic treatment, laparoscopic reimplantation allowed for earlier extubation and resulted in a lower rate of restenosis and hydronephrosis recurrence, with a later onset of these complications. Endoscopic treatment, while offering short‐term benefits, does not permanently resolve ureteral stenosis and is associated with continued decline in renal function and a higher reoccurrence rate of stenosis. Conversely, laparoscopic reimplantation effectively addresses stenosis, allows for earlier removal of the single‐J tube, maintains long‐term ureteral patency, preserves residual kidney function, alleviates hydronephrosis, and reduces the recurrence of restenosis.

Furthermore, the length of the ureteral stricture is crucial in deciding the appropriate surgical approach. Typically, a 1‐cm threshold is used: strictures longer than 1 cm are generally more effectively treated with reimplantation surgery rather than endoscopic methods. For strictures shorter than 1 cm, endoscopic treatment is often preferred. Schöndorf et al. found that minimally invasive endoscopic surgery has a success rate of 50% for strictures less than 1%, but only a 6% success rate for those greater than 1 cm, whereas reimplantation surgery achieves an 86% success rate for larger strictures.^30^ This study did not extensively explore the impact of stricture length due to sample size limitations; however, investigating the effect of stricture length will be a focus of future research.

In previous studies, ureteric reimplantation has been shown to improve renal function in 85.71% of cases, compared to only 20% improvement with balloon dilation.^31^ It has been suggested that surgical exploration of uretero‐intestinal stenosis is advisable when renal function is at risk. In this study, 75% of patients who underwent laparoscopic reimplantation did not develop hydronephrosis, and ongoing follow‐up will assess their long‐term renal function. Although minimally invasive endoscopic techniques have seen improvements, their success rate remains lower than that of reimplantation surgery.^25^ Despite the advantages of minimally invasive procedures, they cannot maintain ureteral patency for extended periods. Therefore, robot‐assisted or conventional laparoscopic reimplantation appears to be a more effective option for managing benign UIAS.

The limitations of the study should be recognized and addressed in future research. The small sample size highlights the need to improve statistical power by increasing the number of surgeries and conducting multicenter collaborative studies to enhance data authority and reliability. Additionally, the study focused on only two treatment groups. Future research should evaluate the efficacy of four different treatment modalities, including endoscopic balloon dilation, endoscopic holmium laser dissection, robotic‐assisted laparoscopic reimplantation, and conventional laparoscopic reimplantation. Furthermore, exploring other treatments for benign UIAS, such as cold knife incision and open surgery, would provide a more comprehensive understanding of the available options.

## 5 CONCLUSION

The management of benign UIAS following RC combined with UD continues to be a critical and challenging area of research in urology worldwide. As surgical techniques and tools advance, exploring more effective treatment modalities becomes essential. With the shift from open surgery to laparoscopic procedures and the growing use of da Vinci robotic surgery, it is vital to compare and analyze the efficacy of laparoscopic reimplantation versus minimally invasive endoscopic surgery to enhance therapeutic outcomes. Endoscopic treatment offers advantages such as shorter operative time, reduced bleeding, faster recovery, and increased safety with lower trauma and economic burden. However, laparoscopic reimplantation is more effective in maintaining long‐term ureteral patency, preserving renal function, and reducing the incidence of restenosis and hydronephrosis, and ultimately yielding superior treatment results.

## AUTHOR CONTRIBUTIONS

**Yixuan Mou:** Writing – original draft (equal). **Cenchao Yao:** Data curation (equal). **Zhenghong Liu:** Methodology (equal). **Pu Zhang:** Methodology (equal). **Xiaolong Qi:** Supervision (equal). **Dahong Zhang:** Supervision (equal). **Yiyang Chen:** Software (equal). **Weiwen Yu:** Writing – review and editing (equal). **Shuai Wang:** Writing – review and editing (equal).

## FUNDING INFORMATION

This project was funded by Medical Science and Technology Project of Zhejiang Province (Grant No. 2024KY702).

## CONFLICT OF INTEREST STATEMENT

The authors declare no conflict of interest.

## ETHICS STATEMENT

The studies involving human participants were reviewed and approved by Zhejiang Provincial People's Hospital and the approval number: QT2022425. The patients provided the written informed consent to participate in this study.

## CONSENT FOR PUBLICATION

Not applicable.

## Data Availability

All data generated or analyzed during this study are included in this published article and available from the corresponding author.
